# Antibacterial, antifungal and antioxidant activity of *Olea africana* against pathogenic yeast and nosocomial pathogens

**DOI:** 10.1186/s12906-015-0941-8

**Published:** 2015-11-17

**Authors:** Peter Masoko, David M. Makgapeetja

**Affiliations:** Department of Biochemistry, Microbiology and Biotechnology, University of Limpopo, Private bag X1106, Sovenga, 0727 South Africa

**Keywords:** *Olea africana*, Antibacterial, Antifungal, Antioxidant, Minimum inhibitory concentration, Bioautography, Leaf extracts, Total activity

## Abstract

**Background:**

*Olea africana* leaves are used by Bapedi people to treat different ailments. The use of these leaves is not validated, therefore the aim of this study is to validate antimicrobial properties of this plant.

**Methods:**

The ground leaves were extracted using solvents of varying polarity (hexane, chloroform, dichloromethane (DCM), ethyl acetate, acetone, ethanol, methanol, butanol and water). Thin layer chromatography (TLC) was used to analyse the chemical constituents of the extracts. The TLC plates were developed in three different solvent systems, namely, benzene/ethanol/ammonium solution (BEA), chloroform/ethyl acetate/formic acid (CEF) and ethyl acetate/methanol/water (EMW). The micro-dilution assay and bioautography method were used to evaluate the antibacterial activity of the extracts against *Escherichia coli*, *Pseudomonas aeruginosa*, *Enterococcus faecalis* and *Staphylococcus aureus* and the antifungal activity against *Candida albicans* and *Cryptococcus neoformans*.

**Results:**

Methanol was the best extractant, yielding a larger amount of plant material whereas hexane yielded the least amount. In phytochemical analyses, more compounds were observed in BEA, followed by EMW and CEF. Qualitative 2, 2- diphenylpacryl-1-hydrazyl (DPPH) assay displayed that all the extracts had antioxidant activity. Antioxidant compounds could not be separated using BEA solvent system while with CEF and EMW enabled antioxidant compounds separation. The minimum inhibitory concentrations (MIC) values against test bacteria ranged between 0.16 and 2.50 mg/mL whereas against fungi, MIC ranged from 0.16 to 0.63 mg/mL. Bioautography results demonstrated that more than one compound was responsible for antimicrobial activity in the microdilution assay as the compounds were located at different R_f_ values.

**Conclusions:**

The results indicate that leaf extracts of *Olea africana* contain compounds with antioxidant, antibacterial and antifungal activities. Therefore, further studies are required to isolate the active compounds and perform other tests such as cytotoxicity. *Olea africana* may be a potential source of antimicrobial compounds.

## Background

The treatment of diseases began long time ago with the use of herbs. They were the only medicinal system before modern or orthodox medicine could be invented [1]. According to the World Health Organisation [2], up to 80 % of the population in Africa use traditional medicine to serve their health needs. Traditional remedies can be derived from any part of the plant, such as barks, leaves, flowers and seeds. These remedies can be prepared from a single plant or a combination of numerous plant species [3]. The mixture could be simple, for example, common known remedies used to treat minor illnesses such as colds, headaches, stomach and menstrual pains. Complex preparations are often used for life threatening diseases [4].

Medicinal plants contain bioactive compounds with the ability to heal. These include saponins, tannins, essential oils, flavonoids, alkaloids and other chemical compounds found as secondary metabolites in plants [5]. Most commercial drugs currently used are of plant origin. The pharmacological history has an endless list of examples such as picrotoxin, aspirin, etc. Potential drugs may be discovered from plants which are recommended regularly and are observed to be effective [6].

Plant secondary metabolites are largely viewed as potential source of novel antibiotics, insecticides and herbicides. This is because of their biological significance and potential health benefits such as antioxidant, anti-aging, anti-atherosclerotic, antimicrobial and anti-inflammatory activities [7]. Regular intake of plant products rich in phenolics have been reported to reduce risks of developing chronic diseases such as cancer, heart diseases and diabetes [8].

When people develop novel drugs to combat diseases, microorganisms also develop new ways to strengthen themselves in order to survive. Nevertheless, plants are capable of developing new natural antimicrobials than mankind remedies [9]. Therefore, plants remain promising for discovery of new bioactive compounds. Although a lot of species have been examined for antimicrobial properties, the vast majority of them have not yet been analysed [10]. Information gathered from ethnic groups or indigenous traditional medicine has played a significant part in the discovery of novel chemotherapeutic agents from plants [11].

The African wild olive, previously known as *Olea africana* subspecies *Cuspidata* is now regarded as *O. europaea* subspecies *africana*. The names depend on which taxonomy and nomenclature are followed. Its common name is African wild olive and vernacular names are umquma (Zulu, Xhosa and Ndebele) and motlhware (Tswana and Sotho). The plant belongs to the family Oleaceae [12]. It is geographically distributed throughout the Southern Africa and Northwards through east Tropical African into Eritrea. It has a variety of habitats, from forest, riverside, open grassveld, bush, mountain kloops and rocky ledges [13].

The *O. europaea* subspecies *africana* plant leaves are used in folk medicine as a remedy for eye infections, sore throat, urinary tract infections, kidney problems and backaches or headaches. It is also used as a hypotensive, emollient, febrifuge and styptic [14]. The leaves of the tree were reported to be potent for the treatment of malaria in 1854 [15].

In this study we are reporting the antibacterial, antifungal and antioxidant activities of *O. europaea* subspecies *Africana*. We have also reported that this species have potential to be used as traditional medicine and furthermore, it can be developed into antimicrobial drugs.

## Methods

### Plant collection

The leaves of *Olea africana* were collected from the University of Limpopo (Turfloop campus), South Africa in Summer, 2013. Plant identity was confirmed by Dr Brownyn Egan (University of Limpopo Herbarium) and the herbarium voucher number was UNIN 11938. The leaves were dried at room temperature. The dried leave material were ground to fine powder using an electric grinder and stored in an air-tight container in a dark place until extraction procedure to prevent oxidation.

### Extraction procedure

The leaves of *O. africana* were dried at room temperature (25 ± 2 °C) for two weeks and grounded to fine powder. The ground leaves (1 g) were extracted with 10 mL of different solvents of varying polarity (hexane, chloroform, dichloromethane, ethyl acetate, acetone, ethanol, methanol, butanol and water) using 50 mL centrifuge tubes. Extraction was performed three times with the same volume of solvent added repeatedly. The extracts were filtered into pre-weighed universal bottles. The filtrates were placed under a fan to evaporate the solvents and the quantity of plant material extracted was determined. The dried crude extract was stored at 4 °C.

### Microorganisms used in the study

#### Bacterial species

The test organisms were supplied by the Department of Biochemistry, Microbiology and Biotechnology section at the University of Limpopo (Turfloop campus). Two Gram positive bacteria (*Staphylococcus aureus* ATCC 29213 and *Enterococcus faecalis* ATCC 29212) and two Gram negative bacteria (*Escherichia coli* ATCC 28922 and *Pseudomonas aeruginosa* ATCC 27853) were used as test microorganisms. These are the major cause of nosocomial infections in hospitals [16] and are primarily the strains recommended for use by the National Committee for Clinical Laboratory Standards [17]. The bacterial species were maintained on nutrient agar at 4 °C. The cells were inoculated and incubated at 37 °C in nutrient broth for 12 h prior to screening tests.

### Fungal species

The isolates of *Candida albicans* and *Cryptococcus neoformans* were used in this study. The isolates were obtained as a donation from University of Pretoria, Faculty of Health Sciences. The fungal species were maintained on sabouraud dextrose agar at 4 °C. The fungal species were inoculated in sabouraud dextrose broth and incubated at 35 °C prior to screening tests.

### Thin layer chromatography (TLC) for Phytochemical analysis

The chemical constituents of the plant extracts were analysed by thin layer chromatography (TLC) using aluminium-backed TLC plates (Merck, silica gel 60 F_254_). The extracted plant material was re-dissolved in acetone to a final concentration of 10 mg/mL and 10 μl of the extract was spotted onto a TLC plate. The TLC plates were developed in solvent systems of varying polarity, i.e. ethyl acetate: methanol: water (10:5.4:4), [EMW] (polar/neutral); chloroform: ethyl acetate: formic acid (10:8:2), [CEF] (intermediate polarity/acidic) and benzene: ethanol: ammonium hydroxide (18:2:0.2) [BEA] (non-polar/basic) [18]. The plates were removed from the tanks, air dried under a fumehood cabinet and observed under ultra-violet (UV) light (254 and 365 nm). To detect compounds which were not visible or fluorescing under UV light, vanillin sulphuric acid reagent (0.1 g vanillin (Sigma ®): 28 methanol: 1 mL sulphuric acid) was used and chromatograms were heated at 110 °C for optimal colour development.

### Phytochemical analysis test of extracts

The following photochemical analysis were performed; reducing sugars [19], anthraquinones [19], terpenoids (Salkowski test) [6], flavonoids [6], saponins [20], tannins [21], alkaloids [22], cardiac glycosides (Keller- Killiani test) [6]. Steroids [6] and phlobatannin [6].

### Qualitative 2, 2-diphenyl-1-pacrylhydrazyl (DPPH) assay on TLC

The plant extracts were separated using TLC as described in Thin layer chromatography (TLC) for Phytochemical analysis. The chromatograms were air dried and sprayed with 0.2 % 2, 2-diphenyl-2-picryl-hyrazyl (DPPH) (Sigma^®^) to detect any antioxidant compounds present in the separated plant extracts. The presence of antioxidant activity was detected by yellow spots against a purple background on TLC plates sprayed with 0.2 % DPPH in methanol [23].

### Antimicrobial activity

#### Quantitative antibacterial and antifungal activity assay by Minimum Inhibitory Concentration (MIC)

The MIC values for bacterial strain were determined using the serial microplate dilution method developed by Eloff [24] while the MIC for fungi was determined using the microplate serial dilution modified by Masoko et al. [25]. Ampicillin (Sigma^®^) and Amphotericin B (Sigma®) were used as positive control for bacterial strains, and fungal strains respectively while acetone was used as the negative control for both. Total activity of the extracts was calculated by dividing the MIC values with the mass extracted from 1 g of the plant material. The resultant value indicate the volume to which the extract can be diluted and still inhibit the growth of the test organism [26].

### Qualitative antibacterial and antifungal activity assay by Bioautography

The bioautography procedure for bacterial strain was done according to Begue and Kline [27], modified by Masoko et al. [28] to screen for compounds with antifungal activity. Clear zones indicated growth inhibition by compounds with antibacterial or antifungal activity.

## Results and discussion

The plant material was extracted using different solvents of varying polarity (hexane, chloroform, dichloromethane (DCM), ethyl acetate, acetone, ethanol, methanol, butanol and water). Majority of traditional healers use water to prepare their decoctions because water is not harmful and is the only solvent available. A major challenge of using water for extraction is that non-polar bioactive compounds cannot be extracted. The type of solvent used in the extraction procedure determines the success of isolating compounds from plant material [29]. Therefore, to extract all compounds it is important to extract using different solvents of varying polarity to cover the polarity range. After evaporating the solvents used for extraction, the extracts were re-dissolved in acetone because acetone has been reported to be harmless towards fungi [30] and bacteria [24].

**Fig. 1:**
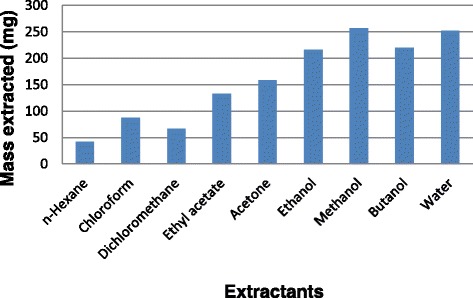
The mass extracted from 1 g of plant dried leaves using different solvents

**Fig. 2:**
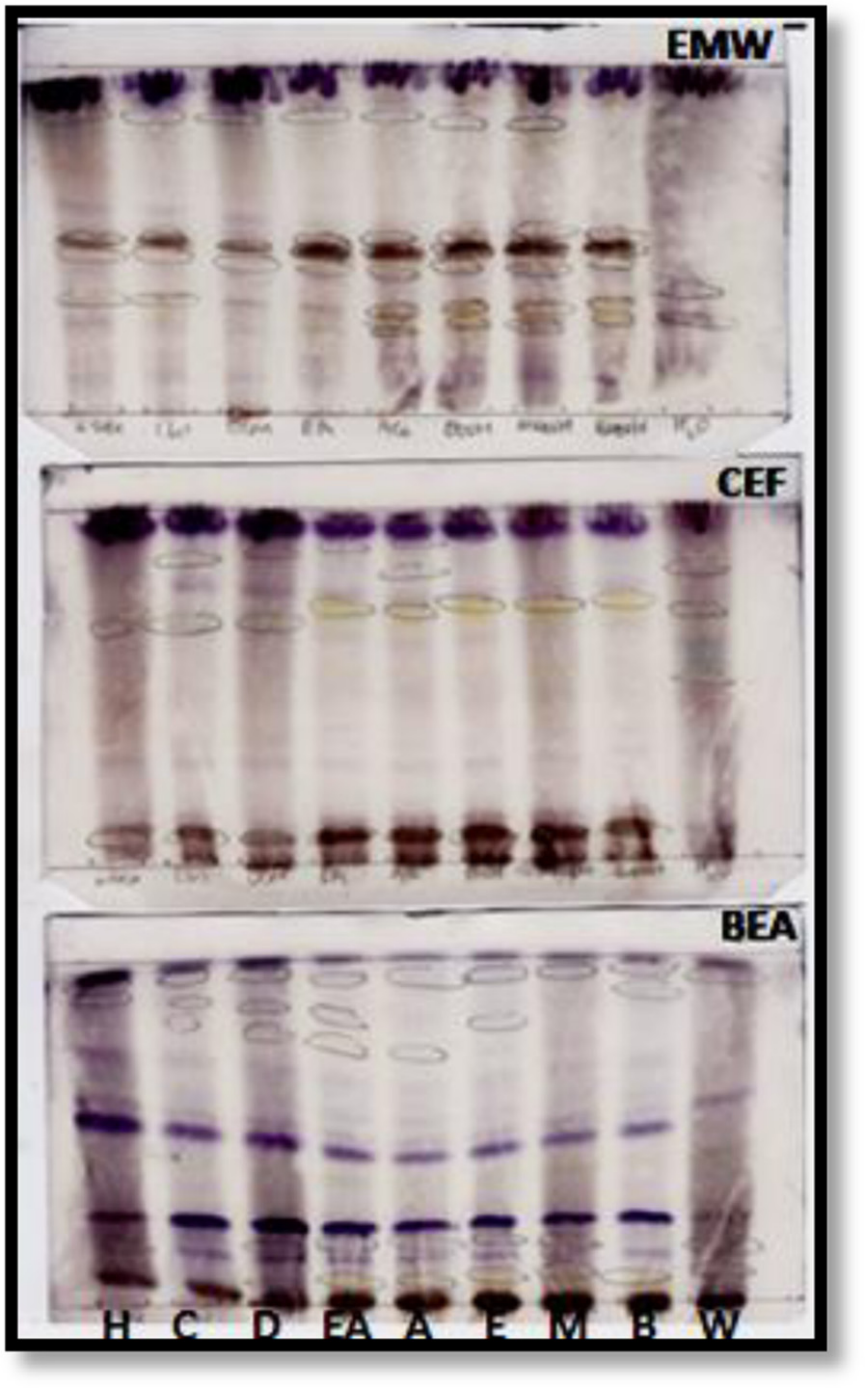
Chromatograms sprayed with vanillin sulphuric-acid reagent to show compounds extracted with hexane (H), chloroform (C), dichloromethane (D), ethyl acetate (EA), acetone (A), ethanol (E), methanol (M), butanol (B) and water (W) from *Olea africana*

Of the nine solvents used, methanol was the best extractant, extracting greater quantity of plant material than the other solvents used (Fig. 1). The chemical constituents of the plant extracts were analysed using Thin Layer Chromatography (TLC). More bands were observed on chromatograms developed in BEA, followed by EMW and CEF (Fig. 2). This shows that the leaves of *O. africana* contain mostly non-polar compounds.

**Fig. 3:**
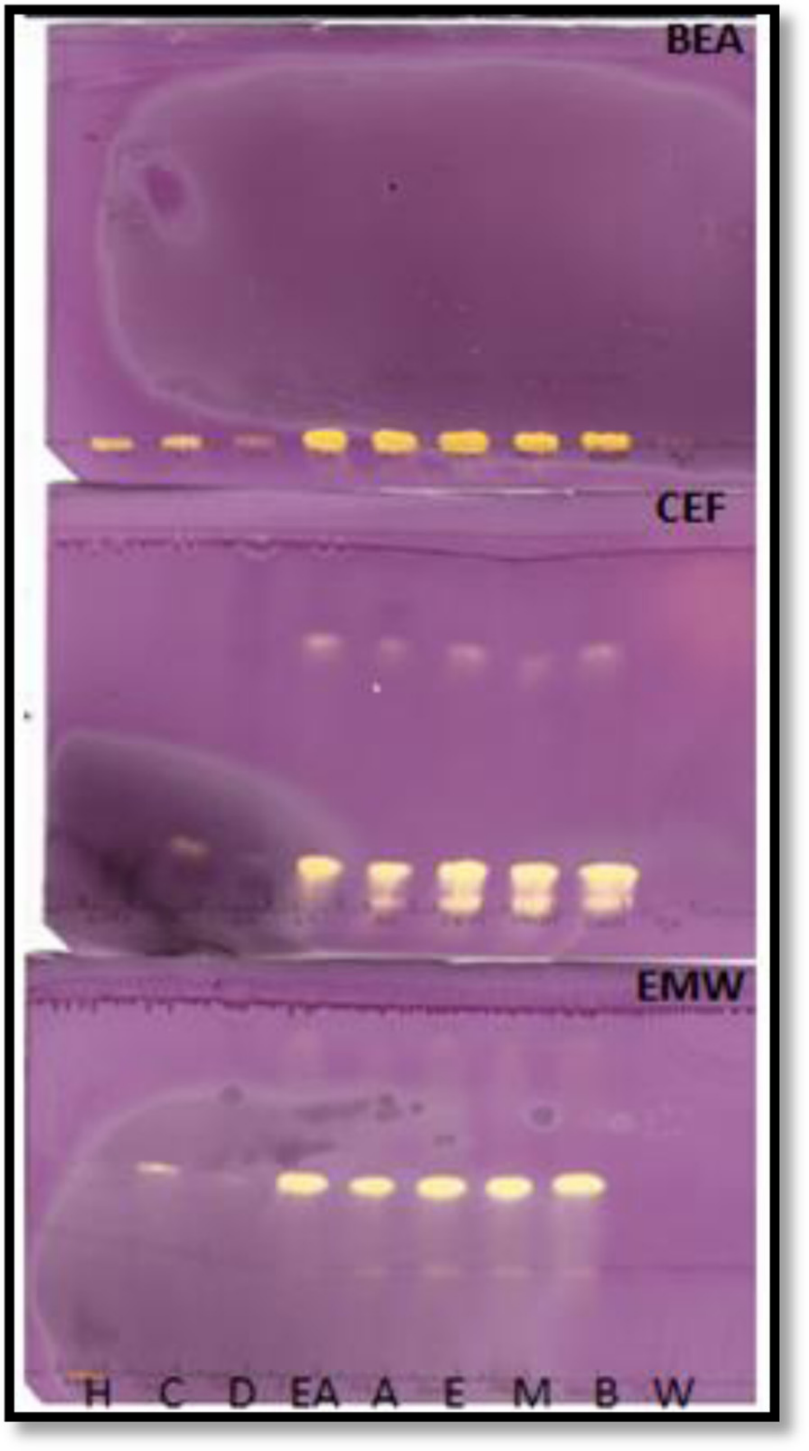
Chromatograms of *Olea africana* extracts separated by BEA, CEF and EMW solvent systems and sprayed with 0.2 % DPPH

This study demonstrated that the leaves of *O. africana* contain compounds with antioxidant activity. This was demonstrated by numerous yellow bands which appeared on chromatograms against a purple DPPH background. Antioxidant compounds were not separated with the BEA solvent system as the yellow bands were observed at the spotted area (Fig. 3). This was probably because the compounds were highly polar to be separated with the non-polar BEA solvent system. The antioxidant compounds were well separated with CEF and EMW solvent systems (Fig. 3). *O. africana* extracts of ethyl acetate, acetone, ethanol, methanol and butanol had compounds with prominent antioxidant activity at retention factor (R_f_) values of 0.13 (CEF) and 0.53 (EMW) respectively. Furthermore, researchers have reported that olive leaf extracts had radical scavenging activity more than twice that of *Camelia sinensis* (green tea) [31].

Table 1 shows the presence of flavonoids, reducing sugar, steroids, tannins and terpenoids and absence of alkaloids, anthraquinones, cardiac glycosides, phlobatanin and saponins.

**Table 1:** Phytochemical constituents of *Olea europaea* subsp *africana* leaves

Constituents	Occurrence
Alkaloids	-
Anthraquinones	-
Cardiac glycosides	-
Flavonoids	+
Phlobatannin	-
Reducing sugar	+
Saponins	-
Steroids	+
Tannins	+
Terpenes/Terpenoids	+

Keys: + = Present, ^_^ = Absent

The MIC values of *O. africana* extracts against *E. coli*, *P. aeruginosa*, *E. faecalis* and *S. aureus* are presented in Table 2. DCM and ethyl acetate extracts were more active compared to the other extracts with MIC values of 0.16 mg/mL against *E. faecalis*. Ethyl acetate and acetone extracts showed prominent activity against all test bacteria with overall average MIC values of 0.30 and 0.32 mg/mL respectively. The water extract had the highest MIC overall average value of 1.74 mg/mL and this shows that the water extract was less active against the four test bacteria. Of all the bacterial pathogens used in this study *E. faecalis* (Gram positive) was the most sensitive test organism with an overall average MIC value of 0.31 mg/mL, followed by *E. coli* (Gram negative) with an overall average MIC value of 0.35 mg/mL. The least sensitive organisms were *P. aeruginosa* (Gram negative) and *S. aureus* (Gram positive) with overall average MIC values of 0.90 and 0.67 mg/mL respectively. The results indicate that activity of *O. africana* extracts is not by targeting the cell membrane. The sensitivity between Gram positive and Gram negative bacteria is frequently attributed to difference in membrane morphology between these organisms [32].

**Table 2:** The MIC values of *Olea africana* extracts in mg/mL against bacteria. The results are the mean of three replicates

Microorganism	H	C	D	EA	A	E	M	B	W	Average	Ampicillin
*E. coli*	0.63	0.31	0.31	0.31	0.31	0.31	0.31	0.31	NA	0.35	0.03
*P. aeruginosa*	1.66	0.52	1.04	0.47	0.42	0.52	0.63	0.73	2.08	0.90	0.02
*E. faecalis*	0.31	0.47	0.16	0.16	0.24	0.31	0.24	0.24	0.63	0.31	0.03
*S. aureus*	1.66	0.26	0.37	0.26	0.31	0.26	0.31	0.26	2.50	0.67	0.008
Average	1.07	0.39	0.47	0.30	0.32	0.35	0.37	0.39	1.74		

*NA* No activity, positive control: Ampicillin was used as positive control. (*H* hexane, *C* chloroform, *D* dichloromethane, *EA* ethyl acetate, *A* acetone, *E* ethanol, *M* methanol, *B* butanol, *W* water)

To determine the total activity of the extracts, the quantity extracted in mg from 1 g of leaf material was divided by the MIC value in mg/mL. Total activity indicate the volume to which the bioactive compounds in 1 g of ground plant material can be diluted and still kill the bacteria or fungi. The extracts with high total activity are considered to be the best for isolation [26]. The results of total activity of *O. africana* extracts against *E. coli*, *P. aeruginosa*, *E. faecalis* and *S. aureus* are presented in Table 3. The results show that the highest total activity was found in methanol extract with the value of 1068 mL against *E. faecalis* while the lowest total activity was displayed by the hexane extract with the values of 25 mL against both *P. aeruginosa* and *S. aureus*. This means that the quantity present in methanol extract can be diluted to 1068 mL but would still inhibit the growth of *E. faecalis*.

**Table 3:** Total activity of the plant extracts in mL

Microorganism	H	C	D	EA	A	E	M	B	W	Average
*E. coli*	67	284	216	428	511	696	827	710	NA	467
*P. aeruginosa*	25	169	64	282	377	415	407	301	121	240
*E. faecalis*	136	187	419	829	660	696	1068	917	401	590
*S. aureus*	25	338	181	510	511	830	827	846	101	463
Average	63	245	220	512	515	659	789	694	208	

*NA* No activity

The MIC values of *O. africana* extracts against *C. albicans* and *C. neoformans* are presented in Table 4. The extracts of chloroform, DCM, ethyl acetate, ethanol and methanol had the same overall average MIC values of 0.16 mg/mL, which was the lowest compared to the other extracts. The water extract had the highest average MIC value of 1.10 mg/mL and this shows that it was less active against the two fungal pathogens used. Overall, both *C. albicans* and *C. neoformans* were sensitive to most of the plant extracts with average MIC values of 0.37 and 0.30 respectively.

**Table 4:** The MIC values of *O. africana* extracts in mg/mL against fungi. The results are the mean of three replicates

Microorganism	H	C	D	EA	A	E	M	B	W	Average	Amph B
*C. albicans*	0.31	0.16	0.16	0.16	0.16	0.16	0.63	0.16	0.63	0.37	0.008
*C. neoformans*	0.16	0.16	0.16	0.16	0.24	0.16	0.31	0.16	0.47	0.30	0.008
Average	0.24	0.16	0.16	0.16	0.42	0.16	0.47	0.16	1.10		

Positive control: Amphotericin B (Amph B) was used as positive control. (*H* hexane, *C* chloroform, *D* dichloromethane, *EA* ethyl acetate, *A* acetone, *E* ethanol, *M* methanol, *B* butanol, *W* water)

Other scientist studied the antimicrobial activities of *O. africana*. Battinellia et al. [33] reported antifungal activity of some aliphatic aldehydes from olive fruit against *Tricophyton mentagrophytes*, *Microsporum canis* and *Candida* spp. Aldehydes tested, inhibited the growth of *T. mentagrophytes* and *M. canis* in the range of concentration between <1.9 and 125 μg/ml. None of the aldehydes exhibited activity against *Candida* spp. Oleuropein isolated from *O. Africana* inhibited the growth of *Salmonella* spp., *Vibrio* spp. and *Staphylococcus aureus* with minimum inhibitory concentration (MIC) between 62.5 and 125 μg/ml for ATCC strains and between 31.25 and 250 μg/ml for clinical isolates. Hydroxytyrosol, derived from oleuropein by enzymatic hydrolysis was reported to have a more broad spectrum and a higher potency by inhibiting *Haemophilus influenzae* and *Moraxella* catharralis; its MIC values were between 0.24 and 7.85 μg/ml for ATCC strains and between 0.97 and 31.25 μg/ml for clinical isolates [34]. Dried leaf extracts (ethanol:water 1:1) at concentrations of 500 mg/ml, were found to be inactive *in vitro* against *Aspergillus fumigatus, A. niger, Fusarium oxysporum, Penicillium digitatum, Rhizopus nigricans, Trichophyton mentagrophytes, Candida albicans* and *Saccharomyces pastorianus* [35]. Furthermore, oleuropein showed activity against several species of *Mycoplasma* [36].

The results of total activity of *O. africana* extracts against *C. albicans* and *C. neoformans* are presented in Table 5. This table shows that the highest total activity was found in butanol extract with the value of 1375 mL against both test fungi. This means that the compound(s) present in the butanol crude extract can be diluted to 1375 mL but would still inhibit the growth of *C. albicans* and *C. neoformans*.

**Table 5:** Total activity of the plant extracts in mL

Microorganism	H	C	D	EA	A	E	M	B	W	Average
*C. albicans*	136	549	419	829	990	1349	407	1375	401	717
*C. neoformans*	264	549	419	829	660	1349	827	1375	537	757
Average	200	549	419	829	825	1349	617	1375	469	

**Fig. 4:**
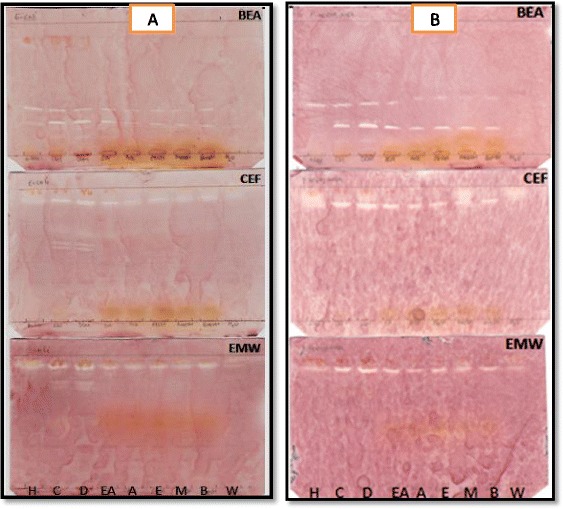
Bioautograms of *Olea africana* extracted with hexane (H), chloroform (C), dichloromethane (D), ethyl acetate (EA), acetone (A), ethanol (E), methanol (M), butanol (B), water (W), separated by BEA, CEF and EMW and sprayed with *E. coli* (**a**) and *P. aeruginosa* (**b**)

**Fig. 5:**
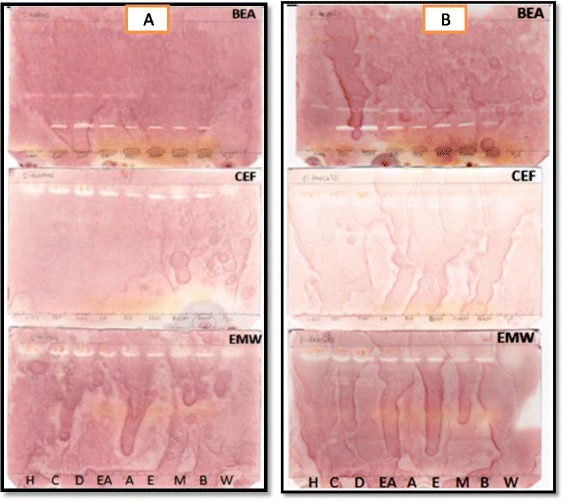
Bioautograms of *O. africana* extracted with hexane (H), chloroform (C), dichloromethane (D), ethyl acetate (EA), acetone (A), ethanol (E), methanol (M), butanol (B), water (W), separated by BEA, CEF and EMW and sprayed with *S. aureus* (**a**) and *E. faecalis* (**b**)

**Fig. 6:**
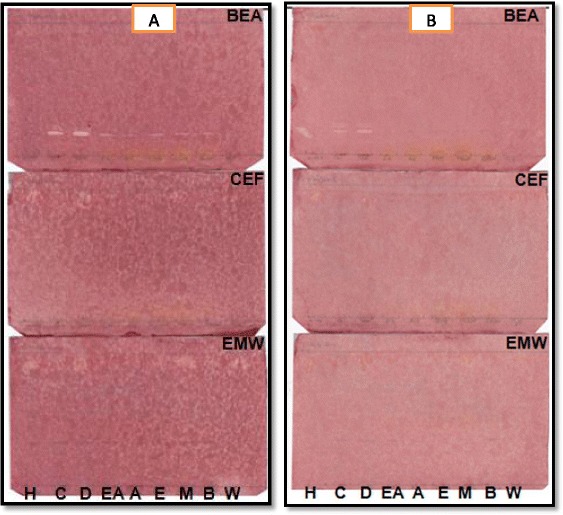
Bioautograms of *O. africana* extracted with hexane (H), chloroform (C), dichloromethane (D), ethyl acetate (EA), acetone (A), ethanol (E), methanol (M), butanol (B), water (W), separated by BEA, CEF and EMW and sprayed with *C. neoformans* (**a**) and *C.albicans* (**b**)

The bioautography assay was used to determine the number of compounds with antibacterial and antifungal activity in the different extracts of *O. africana.* Zones of inhibition were observed by white bands on a purple-red background. These white bands indicated where reduction of INT to the coloured formazan did not take place due to presence of compounds that inhibited the growth of the test bacteria or fungi. The white bands were located at different R_f_ values and this suggested that in the quantitative antimicrobial assays more than one compound was responsible for antimicrobial activity. Bioautography results demonstrated prominent inhibition zones by most extracts of *O. africana* against the growth of the test bacteria (Figs. 4 and 5). The R_f_ values of compounds with antibacterial activity ranged from 0.19 to 0.93. Only the chloroform and DCM extracts separated with BEA displayed antifungal activity against *C. neoformans* (Fig. 6a) and *C. albicans* (Fig. 6b) with R_f_ values of 0.18 and 0.17 respectively. Extracts separated with BEA demonstrated a total of 67 compounds with antimicrobial activity against the test organisms, followed by EMW with a total of 31 compounds and CEF with a total of 25 compounds. This shows that most compounds with antimicrobial activity from *O. africana* leaves are non-polar.

## Conclusion

The results indicate that the leaf extracts of *O. africana* contain compounds with antioxidant activity, antibacterial and antifungal activity. *C. neoformans* and *E. faecalis* were the most sensitive test organisms with overall average MIC values of 0.30 and 0.31 mg/mL respectively. This study indicates that *O. africana* may be a potential source of antibacterial and antifungal compounds. Further studies are required to isolate the active compounds and perform other tests such as cytotoxicity test.

## Abbreviations

TLC: Thin layer chromatography
BEA: Benzene/ethanol/ammonium solution
CEF: Chloroform/ethyl acetate/formic acid
EMW: Ethyl acetate/methanol/water
DPPH: Qualitative 2, 2- diphenylpacryl-1-hydrazyl
MIC: Minimum inhibitory concentrations
ATCC: America type culture collection
INT: *ρ*-iodonitrotetrazolium violet
